# Commentary: Highlighting the need for pesticides safety training in Nigeria: A survey of farm households in Rivers State

**DOI:** 10.3389/fpubh.2022.988855

**Published:** 2022-09-13

**Authors:** Gift Dick Udoh, Jenna L. Gibbs

**Affiliations:** ^1^Norina Farms, Rivers State, Nigeria; ^2^Ag Health and Safety Alliance™, Greenville, IA, United States

**Keywords:** agriculture, personal protective equipment (PPE), pesticides, poisoning, safety, outreach, youth

## Current situation

Agriculture accounts for approximately a quarter of gross domestic product in Nigeria. Common products include dcassava, yam, maize, sorghum, rice, millet, palm oil, cocoa beans and pineapple. Agricultural activities are an important component of peri-urban and rural life in Nigeria, since 70% of households report participation in agriculture and 41% own livestock (1). The entire family—including farm youth— regularly assist with farm activities, including handling of fertilizers and pesticides. This is particularly the case in farming households where women are responsible for production, since they may encourage children and grandchildren to participate and work alongside for both productivity and security reasons (2). These youth are at high risk for exposure to agrochemicals due to their agricultural surroundings and involvement in production. Safe or unsafe handling practices are passed onto these youth at very young ages. Agrochemicals are often purchased at an agricultural supply store (in an open market) and then stored in the home. Previous studies have found that up to 30% of farm households reuse these agrochemical containers for other storage purposes—even for storing food seasonings and palm oil (3, 4). Little is known about socioeconomic factors influencing access to appropriate safety controls for agrochemicals, including the use of personal protective equipment (PPE). This commentary describes current activities being done in Nigeria by Norina Farms to interview agricultural workers in Rivers State to inform development of more community-based, grassroots-style pesticides safety educational programs in the region. The purpose of these interviews was not research driven, rather they were conducted to ensure that the trainings addressed the needs of local farm households.

## Current activities

### Community interviews

In 2, Norina Farms interviewed 152 agricultural workers in Kom-kom, Obeama, Izuoma and Mmiriwanyi Oyigbo in Rivers State, Nigeria. Interviews were conducted in local Pidgin English with the agricultural family inside the home. The homes were selected based on proximity to the Norina Farms office location in Mmiriwanyi. A 10-kilometer geographic radius was identified around the centrally located office, and interviewers contacted all farm households in the region who were available at the time. IRB review for human subjects research was not required since the purpose of the interviews was to identify producer-informed practice solutions to improve a community-based pesticides training program. All agricultural workers (100%) were involved in both cassava and vegetable production, which is common in the region. Interviewers noted demographics, number of youths living in the home, types of pesticides used, and activities related to handling of agrochemicals to inform the training. All the workers reported agricultural youth (age 3 months to 22 years) living in the household.

### Pesticides safety training

In 2018-19 Norina Farms provided a community-based pesticides safety educational program to more than 160 agricultural families in the same region. Agricultural youths as young as 15 years old were present at the training. Educational topics were customized based on the interviews and included (a) sharing stories and discussing adverse health effect experienced as a result of pesticides handling, (b) proper pesticide application methods according to the label, (c) PPE use described on the label and appropriate donning/doffing procedures, (d) proper agrochemicals storage, and (e) container disposal. Personal protective equipment (PPE), including reusable chemical resistant gloves and goggles were provided as incentives for attending the program.

## Current challenges

In Nigeria, many agricultural households are reliant on inorganic fertilizers and pesticides yet have little access to safety controls, such as PPE, described on the label. The interviews conducted by Norina Farms revealed that the most common pesticides used included atrazine, chlorpyrifos, paraquat dichloride, glyphosate, and cypermethrin. Organophosphate pesticides, such as chlorpyrifos, were still reported on crops such as maize, millet, and cassava. This was like another study by Raimi (5), which reported pesticides such as atrazine, cypermethrin, and S-Metolachlor—although the study was limited to inquiring only about specific pesticide types.

Almost none of the agricultural workers (2%) interviewed reported proper use of PPE as stated on the pesticide label. For example, most pesticide labels containing atrazine require coveralls, long sleeves/long pants, and chemical resistant boots and gloves. Chlorpyrifos requires additional eye protection and respiratory protection. Among those interviewed, the only PPE-usage reported were the use of rubber gloves or cloth face coverings. Over half (66%) of participants reported wearing the same clothing in the home after working in the fields, potentially increasing the risk of exposure to others, including any youth, living in the home (6). Ugwu et al. (7) stated that up to 65% of Nigerian farmers reported wearing some form of PPE, with the most common including rubber gloves, protective overalls, and cloth face coverings. Adesuyi et al. (8) found that over 67% of Nigerian farmers near wore some form of PPE with the most common being chemical resistant gloves, hats, and boots. However, both other studies took place in other Nigerian states or near the city of Lagos. The reported use of PPE among this agricultural working population in Rivers State is very low in comparison and further highlights the importance of pesticide safety education efforts in the region.

All families reported little to no training on proper use of safety equipment or personal protective equipment (PPE), with cost and access reported as major barriers. For example, many of the workers did not know how to recognize and request appropriate PPE listed on the label when shopping for agrochemicals at the local agricultural supply store. They indicated that pesticides-related safety equipment is often unavailable at these stores. If PPE, such as chemical gloves, was obtained, several workers stated that it was uncomfortable due to heat or inappropriate sizing. In many cases, the worker stated that the chemical handling gloves were sized too big, making it difficult to handle bottle caps and nozzles. Cloth face coverings continued to be a common substitution for more appropriate respiratory protection. Other studies in Nigeria examine the use of a non-respirator “face mask” or “face covering” so perhaps more research is needed to determine the quality and use of non-respirator face coverings in Nigerian conditions to determine if they are protective or harmful (3, 7, 8).

Almost all agricultural workers stated that they often recycle or reuse agrochemical containers for in-home storage purposes, which seemed to be the case over a decade before the Norina Farms interviews were conducted (3, 4). More recently, in August 2021 a family of 24 individuals, including youth, deceased after consuming ground meal seasoned with fertilizer salt mistaken as food seasoning (9). Most workers also stated that agrochemicals were often stored in the home using unlabeled containers. Therefore, the storage container did not contain listed label ingredients—making it extremely difficult to identify first aid response or proper safety equipment as required by the label.

## Future directions

The interviews conducted by Norina Farms found that agricultural families in Rivers State, Nigeria continue to encounter two major challenges for safe pesticides handling. First, these families have not had much experience using PPE and experience several barriers to obtaining safety equipment. Cloth face coverings continue to be used as a replacement for certified respirators and the same protective clothing is worn in the field and in the home. Only 2% of agricultural workers in the Norina farms interviews reporting using PPE, which was much lower when compared to other state in Nigeria. This highlights tremendous need for more community-based pesticides safety educational programs in this specific region. Second, improper agrochemical storage continues to harm agricultural families since many report reusing the containers for food storage purposes or storing other chemicals in improperly labeled containers. When compared to previous findings, the storage issue seems to be a long-standing problem.

Most pesticides safety training in Nigeria is retailer-based and focuses on larger contract sprayers (10). In this region of Nigeria, we believe that a community-based, grassroots-style pesticides safety program will be more effective than formal trainings focused on adult handlers only. This style of education would involve a family-oriented approach and should be amended for in-person presentation without internet access in rural areas. Even if agricultural workers cannot read or interpret pesticide labels, they should be able to recognize pesticides containers and have access peer-to-peer mentoring if they have questions. If future pesticides safety educational programs focus on raising awareness about proper pesticide labeling and PPE specifications, these agricultural producing families may become more empowered to advocate for PPE at their local agricultural supply store.

## Author contributions

GU of Norina Farms is responsible for the conception of the article, community interviews, and pesticides safety trainings in Nigeria. JG assisted with the writing, organization, and formatting of this article for publication. All authors contributed to the article and approved the submitted version.

## Funding

Funding for this commentary provided by the Ag Health and Safety Alliance™, a 501(c)3 non-profit organization.

## Conflict of interest

Author GU was employed by Norina Farms. The remaining author declares that the research was conducted in the absence of any commercial or financial relationships that could be construed as a potential conflict of interest.

## Publisher's note

All claims expressed in this article are solely those of the authors and do not necessarily represent those of their affiliated organizations, or those of the publisher, the editors and the reviewers. Any product that may be evaluated in this article, or claim that may be made by its manufacturer, is not guaranteed or endorsed by the publisher.
